# IMMerge: merging imputation data at scale

**DOI:** 10.1093/bioinformatics/btac750

**Published:** 2022-11-22

**Authors:** Wanying Zhu, Hung-Hsin Chen, Alexander S Petty, Lauren E Petty, Hannah G Polikowsky, Eric R Gamazon, Jennifer E Below, Heather M Highland

**Affiliations:** Division of Genetic Medicine, Department of Medicine, Vanderbilt Genetics Institute, Vanderbilt University Medical Center, Nashville, TN 37232, USA; Division of Genetic Medicine, Department of Medicine, Vanderbilt Genetics Institute, Vanderbilt University Medical Center, Nashville, TN 37232, USA; Division of Genetic Medicine, Department of Medicine, Vanderbilt Genetics Institute, Vanderbilt University Medical Center, Nashville, TN 37232, USA; Division of Genetic Medicine, Department of Medicine, Vanderbilt Genetics Institute, Vanderbilt University Medical Center, Nashville, TN 37232, USA; Division of Genetic Medicine, Department of Medicine, Vanderbilt Genetics Institute, Vanderbilt University Medical Center, Nashville, TN 37232, USA; Division of Genetic Medicine, Department of Medicine, Vanderbilt Genetics Institute, Vanderbilt University Medical Center, Nashville, TN 37232, USA; Division of Genetic Medicine, Department of Medicine, Vanderbilt Genetics Institute, Vanderbilt University Medical Center, Nashville, TN 37232, USA; Department of Epidemiology, Gillings School of Global Public Health, University of North Carolina at Chapel Hill, Chapel Hill, NC 27514, USA

## Abstract

**Summary:**

Genomic data are often processed in batches and analyzed together to save time. However, it is challenging to combine multiple large VCFs and properly handle imputation quality and missing variants due to the limitations of available tools. To address these concerns, we developed IMMerge, a Python-based tool that takes advantage of multiprocessing to reduce running time. For the first time in a publicly available tool, imputation quality scores are correctly combined with Fisher’s z transformation.

**Availability and implementation:**

IMMerge is an open-source project under MIT license. Source code and user manual are available at https://github.com/belowlab/IMMerge.

## 1 Introduction

Aggregating imputed genetic data is often a challenge in human genetic studies. Imputed genetic data are regularly combined from projects performed in stages or from different studies that are merged to improve power. Additionally, because genetic imputation involves substantial computational resource usage, local and even public server-based imputation of individual datasets is subject to a sample size cap due to computational constraints, requiring large-scale data to be split into multiple batches.

Specifically, two commonly used imputation resources, the TOPMed Imputation Server and the Michigan Imputation Server (Das *et al.*, 2016; Fuchsberger *et al.*, 2015), limit the number of genetic samples that can be imputed per submission. To date, 2686 users are registered on the TOPMed Imputation Server and more than 31.5 million genomes have been imputed (Taliun *et al.*, 2021). The TOPMed sequence data are not widely available at present, so researchers who utilize this reference must submit their data to the publicly available TOPMed imputation server, which has capped the maximum sample size per batch at 25 000 (Michigan Imputation Server, 2020https://mobile.twitter.com/umimpute/status/1357139156464521216). Imputation to the Haplotype Reference Consortium on the Michigan Imputation Server is also capped at 110 000. However, many datasets now exceed these limits. For example, the UK Biobank comprises ∼450 000 genotyped individuals, BioVU of Vanderbilt University Medical Center which has collected 275 000 DNA samples with over 110 000 genotyped individuals, the Colorado Center for Personalized Medicine’s biobank comprises DNA on more than 90 000 individuals, and myCode which comprises 176 000 genotyped individuals.

To work around these limits, researchers divide large datasets into smaller subsets, impute each subset, and merge imputed subsets together. Software such as VCFtools and BCFtools was developed as part of the SAMtools package to parse and manipulate VCF files when this format was first introduced to the public (Danecek *et al.*, 2011; Li, 2011). VCFtools can combine VCF from distinct genomic sites from a common set of individuals but cannot combine data from distinct sample sets. BCFtools is slow due to poor utilization of multiprocessing, does not have flexible options for handling variants that are missing from one or more batches, and does not correctly compute combined imputation quality statistics.

To address these challenges we developed IMMerge, a Python-based tool that can be applied either via the command line or as a Python module. IMMerge is designed to (i) rapidly combine sets of imputed data through multiprocessing to accelerate the decompression of inputs, compression of outputs and merging of files; (ii) preserve variants not shared by all subsets; (iii) combine imputation quality statistics and detect significant variation in single nucleotide polymorphism (SNP)-level imputation quality; (iv) manage samples duplicated across subsets; (v) output relevant combined summary information including allele frequency (AF) and minor allele frequency (MAF) as weighed means, maximum and minimum values.

## 2 Implementation

IMMerge works with Python 3.7 and higher using publicly available packages pandas (version 1.3.3), xopen (version v1.4.0) and command line bgzip. It can be executed as a stand-alone tool or imported and accessed from Python as a module.

### 3.1 Input files and preparation

Minimal preparation is required for files directly output from the TOPMed imputation server. IMMerge requires gzip or bgzip compressed genotype file (*.vcf.gz) and corresponding information file (*.info.gz). The input files must contain a metric of imputation quality, MAF, AF and genotyped status in the INFO column. If merging VCF files from other sources, the preprocessing step will generate the required *.info.gz file from the VCF; example inputs are provided with IMMerge. The input VCF files must be separated by chromosome with variants sorted by position. Paths to input files can be specified as parameters, otherwise, genotype and information files should share the same file root name and be in the same directory. Since the merging operation will copy input data to output files, the working environment must have sufficient disk space to write complete output files.

### 3.2 Select variants and calculate combined allele frequency and imputation quality score

For the first time in a publicly available tool, imputation quality scores are combined using Fisher z-transformation (Silver and Dunlap, 1987). The combined imputation quality, minimum, maximum quality scores and minimum, maximum and weighted mean alternative AF and MAF are provided in the resulting info file. Individuals with missing variants are not counted when calculating combined imputation quality scores. Genotyped status is provided as ‘all’ (all batches genotyped), ‘some’ (at least one batch genotyped) or ‘none’ (no batches genotyped).

IMMerge first generates lists of excluded and retained variants from information files (.info.gz) and writes them in separate output files (*excluded.info.txt and *retained.info.txt). A user-specified combined imputation quality score threshold can be employed to exclude variants. The user-specified option ‘missing’ defines the handling of variants not shared by all input files, so that variants missing from more than a given number of input files can be removed for all samples. Variants kept will have values of ‘.|.’ for all samples in the missing batches.

### 3.3 Merging input files

**Fig. 1.**
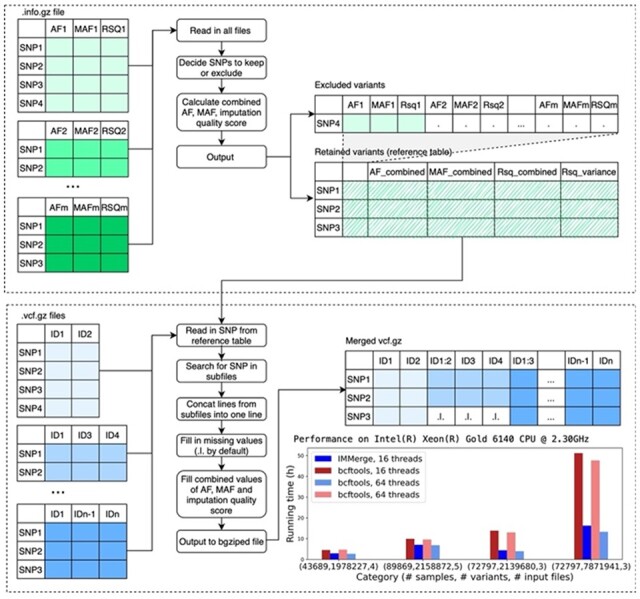
Overview of IMMerge implementation and performance comparison with bcftools on a testing dataset

Input files are then opened simultaneously and read through line by line. Specifically, for the first variant in the retained file, IMMerge searches through an input VCF file by line until it finds the given variant in the retained file or fills in missing values if the variant is not available (as indicated by missing variant information for the batch). Then, IMMerge moves to the next input VCF file and repeats the search process. This is repeated for the next variant in the retained file with the search starting where it previously stopped. Values from all input files are pieced together for output as shown in Figure 1. Duplicated samples will be retained with different IDs and duplicated IDs written to ‘duplicates.txt’. MAF and imputation quality in the INFO column will be replaced by combined values calculated in Step 2.

### 3.4 Outputs

IMMerge outputs four files: merged genotype file (.vcf.gz), two information files which include combined and batch-specific AF, MAF, imputation quality and genotyped status for variants retained or excluded (*excluded.info.txt and *retained.info.txt) and a log file.

## 3 Results

We tested the performance of IMMerge by merging three to five MEGA array sequencing vcf files containing 2420 to 24 300 individuals. In all scenarios, IMMerge completed the merge more quickly than bcftools (Fig. 1). The biggest improvement was seen when combining 72 797 individuals, with a combined 7 871 941 variants, from three VCF files. This took 16.3 h in IMMerge in comparison to 51 h in bcftools, with each tool using 16 threads. While sample size limitations prohibit combined imputation in very large datasets, IMMerge enables researchers to combine genetic data in a computationally efficient manner. This facilitates joint analysis of datasets which increases statistical power, allowing for modeling relationships and population structure across the entire sample such as SAIGE (Zheng and Davis, 2021; Zhou *et al.*, 2018), GENESIS (Gogarten *et al.*, 2019), STAAR (Gaynor *et al.*, 2022; Li *et al.*, 2020) and STAARpipeline (Li *et al.*, 2022).

## Data Availability

There are no new data associated with this article.
